# The Impact of Individual Biases on Consensus Formation

**DOI:** 10.1371/journal.pone.0058989

**Published:** 2013-05-28

**Authors:** Marta Sales-Pardo, Daniel Diermeier, Luís A. Nunes Amaral

**Affiliations:** 1 Dept. d'Enginyeria Química, Universitat Rovira i Virgili, Tarragona, Spain; 2 Dept. of Chemical and Biological Engineering, Northwestern University, Evanston, Ilinois, United States of America; 3 Kellogg School of Management, Northwestern University, Evanston, Ilinois, United States of America; 4 Northwestern Institute on Complex Systems, Evanston, Ilinois, United States of America; 5 Canadian Institute for Advanced Research, Toronto, Canada; 6 Howard Hughes Medical Institute, Chevy Chase, Maryland, United States of America; Northeastern University, United States of America

## Abstract

Social groups of interacting agents display an ability to coordinate in the absence of a central authority, a phenomenon that has been recently amplified by the widespread availability of social networking technologies. Models of opinion formation in a population of agents have proven a very useful tool to investigate these phenomena that arise independently of the heterogeneities across individuals and can be used to identify the factors that determine whether widespread consensus on an initial small majority is reached. Recently, we introduced a model in which individual agents can have conservative and partisan biases. Numerical simulations for finite populations showed that while the inclusion of conservative agents in a population enhances the population's efficiency in reaching consensus on the initial majority opinion, even a small fraction of partisans leads the population to converge on the opinion initially held by a minority. To further understand the mechanisms leading to our previous numerical results, we investigate analytically the noise driven transition from a regime in which the population reaches a majority consensus (efficient), to a regime in which the population settles in deadlock (non-efficient). We show that the mean-field solution captures what we observe in model simulations. Populations of agents with no opinion bias show a continuous transition to a deadlock regime, while populations with an opinion bias, show a discontinuous transition between efficient and partisan regimes. Furthermore, the analytical solution reveals that populations with an increasing fraction of conservative agents are more robust against noise than a population of naive agents because in the efficient regime there are relatively more conservative than naive agents holding the majority opinion. In contrast, populations with partisan agents are less robust to noise with an increasing fraction of partisans, because in the efficient regime there are relatively more naive agents than partisan agents holding the majority opinion.

## Introduction

An intriguing feature of social groups is the ability of interacting agents to efficiently coordinate in the absence of a central authority. The widespread availability of social networking technologies has increased the success and impact of decentralized coordination, as e.g. during the “Arab Spring” [1] or the Spanish protests in 2011 that simultaneously started in 60 different towns known as the 15M movement [2]. Interestingly, decentralized coordination arises in spite of the heterogeneities across the individuals comprising these social systems and the initial opinions held by those individuals [3], [4]. Individual and group biases, however, can polarize public opinion on controversial matters, undermining society's ability to reach effective policy solutions [5], [6] and often completely changing the dynamics of the process [7], [8]. Thus, a fundamental question is how individual biases affect the efficiency of the collective in reaching consensus.

To explore these questions, we recently introduced a model in which agents may have conservative or partisan biases toward one of the two possible opinions. These agents update their opinions using a modified local majority rule that takes into account the potential effect of noise in the communication channel and the personal bias of each agent [9], [10]. In this model, the noise in the communication channel accounts for any external factor that might lead to an agent misinterpreting another agent's opinion, for instance an ambiguity in the conversation or a simple misunderstanding.

Numerical simulations for finite populations show that when the population is exclusively composed of naive agents, i.e. agents that follow a simple majority rule, the population can reach consensus on the initial majority opinion in the presence of noise [9]. Interestingly, including conservative agents who have a bias toward their current opinion enhances the population's efficiency in reaching consensus when noise is present, whereas even a small fraction of partisan agents who have a bias toward the opinion opposite to the one held by the majority of the agents, would be enough to draw the population into the consensus on the opinion of the minority.

Here, we investigate the model analytically using a mean-field approximation and show that results from model simulations can be interpreted in terms of the stability of the efficient steady-state solution of the model, i.e. the regime in which a majority of the population converges to the same opinion. We distinguish two cases: i) agents with no opinion bias, in which agents do not have an *a priori* bias toward one of the two opinions and where it turns out to be a continuous transition along the noise axis from an efficient regime to a regime in which the population settles into deadlock; ii) agents with an opinion bias, in which agents have a bias toward a specific opinion and as a result there is a discontinuous transition between an efficient regime and a partisan regime which draws the population into a consensus opposite to the opinion held initially by the majority of the agents. Interestingly, in the latter case, the noise amplitude at which the transition occurs depends on the initial density of agents holding the majority opinion. Furthermore, the analysis of the steady-state solution close to the transition point can shed light onto the mechanisms responsible for the higher robustness of systems of agents with an increasing fraction of conservatives against noise amplitude and the decrease in robustness of a population with an increasing fraction of partisans.

### Background

The theoretical study of consensus formation in a population has received attention in the modeling community, especially because simple spin models already display consensus formation in a similar way to that observed in real social systems. In such systems, agents(spins) can hold two possible opinions 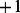 or 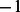 and update their opinions according to some rule. Moreover, the opinion update rules in these models can be easily modified to mimic real situations. The most studied of these models is the voter model [11], [12] in which at each step, an agent picks a neighbor at random and updates her opinion to the opinion of the neighbor. The mean field approximation predicts that this model has two absorbing solutions in which the whole population adopts the same opinion 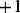 or 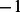. If the network of connections between agents has the topological features present in empirical social networks – high-clustering and the small-world property – then, a finite population reaches consensus in a shorter time than in regular lattices [13]–[16]. However, if one organizes the population into loosely connected topological communities, then each community may reach their own independent consensus [17], [18].

With the aim of depicting more realistic dynamics, studies in the literature have incorporated a number of features such as majority update rules that resemble more how the social environment influences individual preferences [9], [10], [19]–[24], or the presence of noise in the information transfer channel [9]–[11], [25], which is critical for the system to reach consensus.

Among the features with the largest impact in the system's dynamics is the introduction of agent bias [8], [10], [20], [22], [26]–[28]. Differently to other studies, our model [10] considers agents that have a bias with strengths that can vary and introduces noise in the channel of information transfer between agents. The introduction of conservative agents can help the population in the coordination task without the need of a central authority, whereas the inclusion of even a very small fraction of partisan agents prevents the system from coordinating.

Our interest is to study whether a small initial opinion imbalance toward one opinion ends up in the strengthening of that opinion (*efficient regime*), deadlock (*non-efficient regime*), or in the majority of agents adopting the opinion held initially by a minority of partisans (*partisan regime*). Because the steady-state solution of the model does not necessarily correspond to a pure consensus in which all the agents converge toward the same opinion, we look at the *efficiency* or capacity of the population to amplify an initially small majority. In particular, we study the transition between the different regimes in the mean-field approximation and show how the observed phenomena in numerical simulations can be understood in terms of the set of steady-state solutions to the model when agents with different biases are present in the population. For some cases, we provide the analytical solution for the transition line and characterize the effect of the fraction of non-naive agents on the coordination efficiency when we vary the noise amplitude in the communication channel.

## Results

### Model

Consider a population of 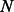 agents that hold binary opinions 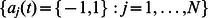. Further, assume that each agent has 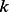 neighbors and a preference toward a specific state 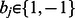. We define two broad classes of agents: “well-intentioned” agents who prefer the opinion they currently hold, so that 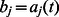, and “partisan” agents who have a fixed preferred opinion so that 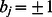. Each agent also has a bias strength 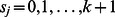 toward her preferred opinion. 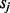 specifies the agent's ability to counter peer pressure, that is, 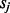 specifies how strong the social pressure of the social neighborhood needs to be for the agent to adopt his non-preferred opinion. If 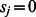, then a well-intentioned agent is *naive*, otherwise the agent is *conservative*. As a result, while all types of agents may change their state in response to peer pressure, a partisan agent will defect back to his preferred state if peer pressure decreases below a threshold value.

At each time step, agent 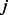 takes into account his own opinion and the current opinion of his 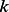 neighbors and updates his opinion following a generalized majority rule that depends on 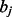 and 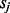,
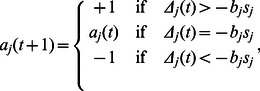
(1)where,



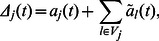
(2)and 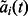 is the perceived opinion of neighbor 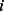 and 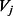 is the set of neighbors of agent 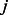. Because the communication channel between agents is noisy, with probability 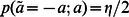 agent 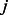 perceives the opinion of neighbor 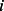 as being the opposite to the one he currently holds. Note that if the sum of perceived opinions of the agent's neighbors overcomes the strength of his bias to a specific opinion, then the agent will defect from his preferred opinion.

### Mean-field approximation

Our goal is to find the steady-state efficiency of the system



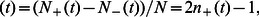
(3)where 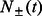 is the number of agents holding opinion 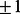 at time 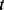, and 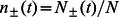. In what follows, we denote 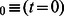 and 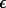 as the steady-state value of the efficiency.

At each time step 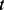, agent 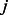 will change opinions at a rate 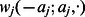, where  expresses de dependency of the rate on 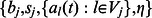. The expected change in 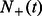 is then
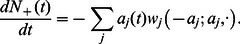
(4)


The next step is to find an expression for 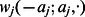. Assume we pick an agent 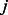 at random. In the mean-field approximation, agent 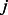 is surrounded by an *average neighborhood* in which each neighbor holds opinion 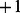 with probability 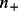 and opinion 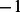 with probability 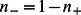. Thus, the probability 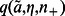 of agent 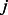 perceiving a neighbor holding opinion 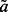 is the same for all agents,
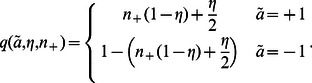
(5)


Equation (5) enables us to obtain the probability 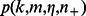 that an agent with 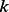 neighbors will perceive 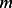 neighbors holding opinion 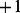 and 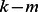 neighbors holding opinion 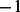


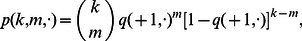
(6)where we have used that 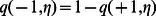.

Consider an agent holding opinion 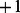. From Eq. (1), we see that for a well-intentioned agent (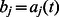) with bias strength 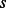 to change her opinion to 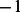, she needs to perceive at least 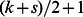 agents holding the opposite opinion 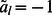. The rate at which a well-intentioned agent changes opinions is thus
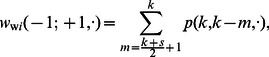
(7)where 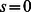 corresponds to a naive agent and 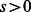 corresponds to a conservative agent (see Table 1).

**Table 1:** Summary of transition rates for each agent type.

agent type 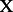	bias strength	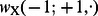	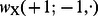
naive	*s* = 0	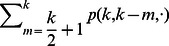	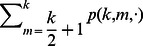
conservative	*s* = *s*	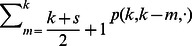	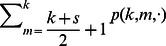
neg. partisan	*s* = *s*	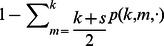	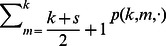
pos. partisan	*s* = *s*	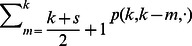	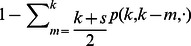

Note that, for a positive partisan (that is, 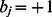) holding opinion 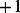, the rate of changing opinions is that of a well-intentioned agent with the same bias strength 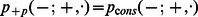. A negative partisan (that is, with 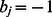), however, will always adopt opinion 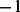, unless he perceives 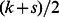 agents as holding opinion 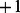. Thus, the rate at which a negative partisan holding opinion 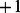 changes opinions is
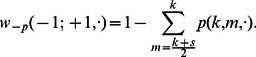
(8)


Similar rules apply for agents changing from opinion 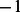 to opinion 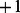 (see Table 1).

Consider a mixed population of 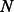 agents in which there are 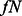 non-naive agents with a fixed bias strength 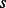 and 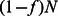 naive agents. Further assume that 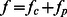, where 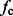 is the fraction of conservative agents and 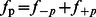 is the total fraction of partisan agents, both negative and positive. Because agents do not change type, that is 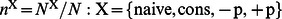 is constant, and the rate of change depends on the type of agent 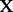, we can rewrite Eq. (4) as follows
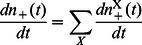
(9)

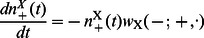


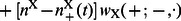
(10)


A steady-state solution to Eq. (10) is one that satisfies 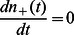. In the following, we distinguish two families of solutions: symmetric and non-symmetric.

### Agents with no opinion bias: conservatives and equal fractions of negative and positive partisans

Consider a population comprised entirely of well-intentioned agents i.e., either naive (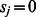) or conservative (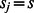) agents with a bias toward the opinion they currently hold. Because there is no *a priori* bias against a particular opinion, the solution 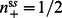 is always a solution to the steady state condition 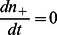. It is easy to check that the zero efficiency solution  is a solution to both naive and conservative populations and is thus a solution of a system comprising both. However, this solution is not stable for low values of noise.

For simplicity, let us consider a population of well-intentioned agents of the same type and assume that 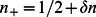 with 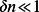. Straightforward algebra yields
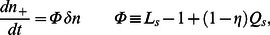
(11)where 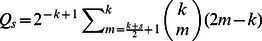 and 
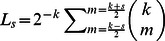
.

**Figure 1:**
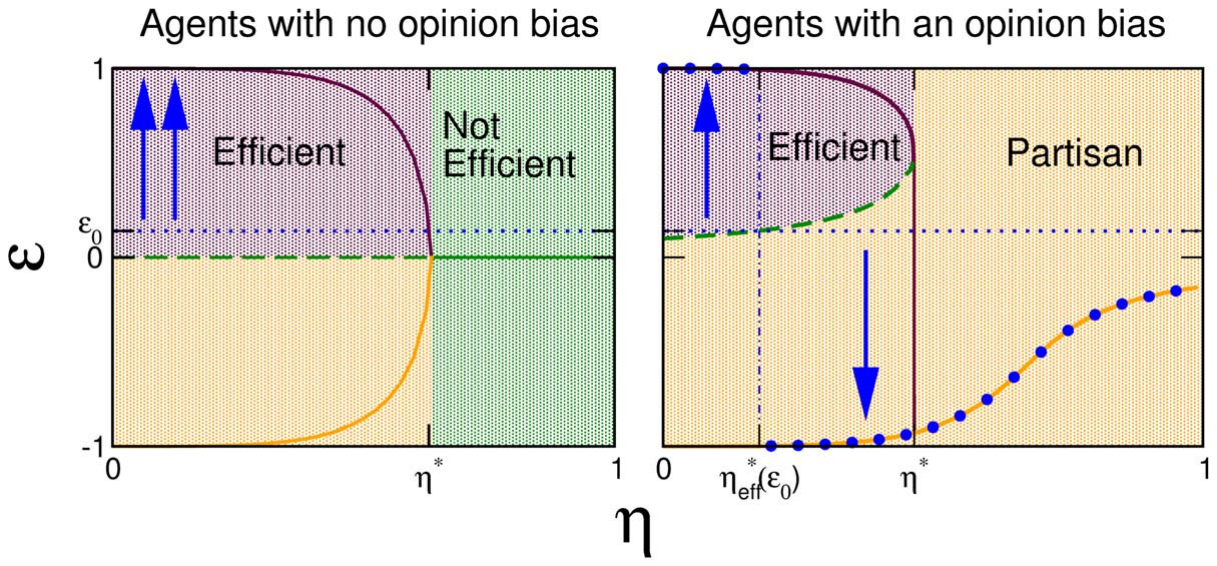
Mean-field solution for a mixed population of agents. For a fixed fraction 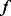 of non-naive agents we plot the steady-state efficiency 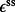 versus the noise amplitude 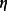. We consider two scenarios: (a) *agents with no opinion bias* and (b) *agents with an opinion bias* (see text). Continuous lines show stable solutions, and discontinuous lines show unstable solutions. The maroon regions show the set of initial conditions 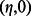 for which 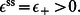 The orange region covers 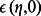 for which 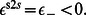 The green region covers initial conditions 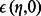 for which 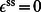. In (b), blue dots show 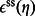 for the initial condition indicated by the dotted blue line. Blue arrows indicate the steady-state solution that corresponds to the initial condition represented by the blue dotted line.

The condition 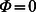 yields the value 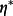 at which the transition between efficient and non-efficient regimes occurs, 
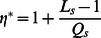
. For 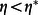, 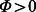 and the solution 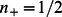 is not stable. In this regime, there are two stable symmetric solutions for 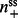, one such that 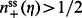 that yields an efficiency 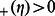, and another one such that 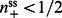 that yields an efficiency 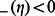 (Fig. 1). As 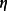 increases, these two solutions approach the unstable solution 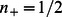. For 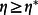, all solutions merge, and there is only one stable solution for the efficiency 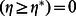. Therefore, in the mean-field approximation, a system comprised of naive agents with an initial condition of 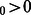 (see dotted blue line in Fig. 1), will transition from an efficient regime (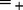) for 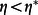, to an inefficient regime (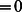) for 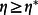.

**Figure 2:**
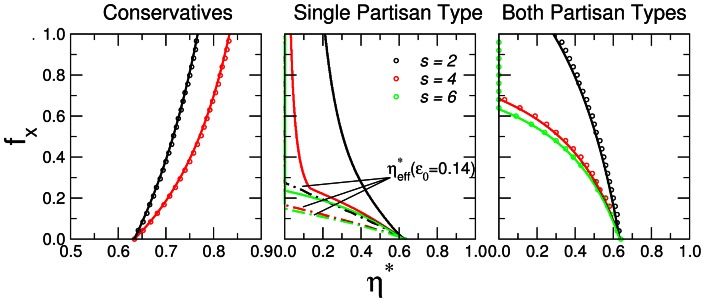
Transition line for the mean-field solution for a mixed population of agents with. 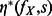
 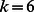 We plot the noise amplitude 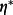 at which the positive efficiency solution ceases to be stable (see Fig. 1) for a fraction 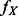 of non-naive agents and for different bias strengths 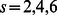 (see text for the conservative case and 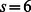). Solid lines show the numerical solution for the steady state condition and dots show the theoretical values from Eqs. (12) (conservatives) and (14) (both types of partisans). For the symmetric cases, those with *conservatives* and both types of *partisans*, the line separates the efficient regime for low values of noise amplitude from the non-efficient regime. In the case in which there are only negative partisans, the line separates the efficient regime from the partisan regime in which the system is drawn into a consensus on the opinion held by the initial minority opinion. Note how in the symmetric case 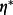 is independent of 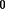, the efficiency of the system at time zero. However, in the non-symmetric case of only negative partisans, the transition line depends on the initial efficiency 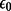. As a guide we show transition lines for 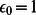 (solid lines) and 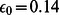 (dotted lines).

One can generalize this result to a mixed population of 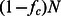 naive agents and 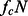 conservative agents with fixed bias strength 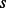. Because 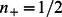 is a steady state solution for both kinds of agents, we assume that the solution can be written as 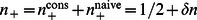 with 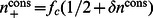 and 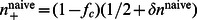. We obtain the following transition line 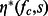 (see Methods for a derivation)
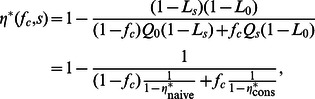
(12)where 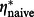 and 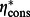 are the solutions for the case with a single type of well-intentioned agents for 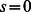 and 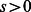, respectively (see Fig. 2).

Note that, because 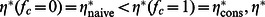 grows with the fraction of conservatives, indicating that the presence of conservatives makes the system more robust to perturbations for all 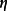 (Fig. 2). In fact, if we look at the relative fraction of agents of each type in excess of 1/2, 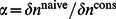 (see Methods), we find that
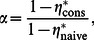
(13)which shows that for 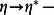, close to the transition between efficient and inefficient regimes, there are more conservative agents holding the majority opinion than naive agents.

Note that for 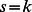 the system is not symmetric. Because partisan agents settle into their preferred opinion, if there is a bias in the initial distribution of opinions (that is, 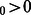), then, the efficiency of the system is always positive.

### Agents with an opinion bias: negative partisans

Because partisans have a preference toward a fixed opinion, 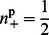 is not a solution of 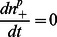, thus there is no stable non-efficient regime for high noise amplitudes. For the case in which there is a small fraction of negative partisans 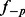, for low values of noise, there are still two stable 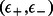 steady-state solutions with positive and negative efficiencies (Fig. 1), and an unstable solution with 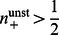. At 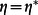 the positive efficiency solution merges with the unstable solution and becomes marginally stable. Thus, for 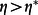 the only stable solution is a “partisan” solution in which the majority of agents adopt the opinion of the partisan minority regardless of the initial majority opinion.

**Figure 3:**
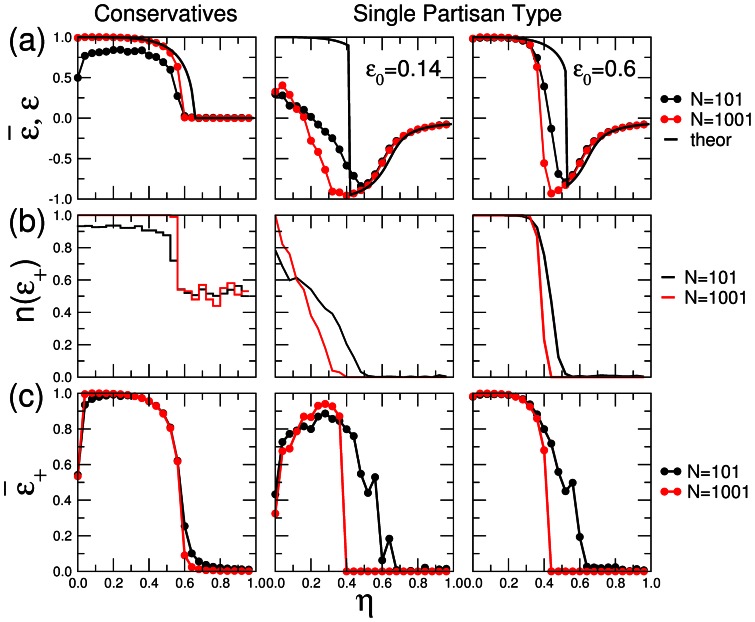
Comparison between model simulations for finite system sizes and the mean-field results. We consider populations of naive agents with 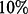 of conservatives or negative partisan agents with bias strength 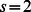. We build a network following the model proposed by Watts and Strogatz [30] using the same parameters as in [10]–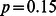 and 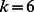. Once the system has reached a steady-state we obtain: (a) 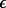, the average efficiency, (b) 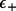, the average efficiency for the realizations with positive efficiency, and (c) 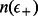 the fraction of realizations in which  is positive. We show results for populations of 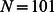 (black) and 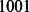 (red) agents and 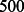 and 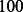 realizations, respectively. In the top row, solid black lines indicate the solution to the mean-field model 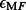. In the case of negative partisans, we show results for two initial conditions 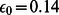 and 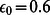. Note how in the case of conservative agents there is a good agreement between simulations and the mean-field expectation. There is a transition from a regime in which almost all realizations have 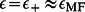 to a regime for 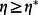, in which the population ends half of the time with 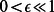. In the case with partisan agents, we show how the initial condition affects the behavior of the system as predicted. Note how there is a transition between a region in which some realizations have 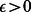, to a partisan regime in which no realizations have 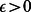 and the efficiency is very similar to that of the mean-field approximation.

For a population with an initial majority holding opinion 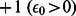 there is thus a discontinuous transition from an efficient regime to a partisan regime. The transition point 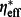 depends on 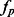 and 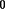; when 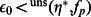 (see crossing between dotted blue line and green line in Fig. 1b), then the population transitions into the partisan regime. Interestingly, 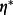 decreases with 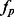 and for 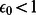 there is a finite 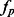 at which the system can no longer be efficient (Fig. 2b). This finding is in agreement with the results for numerical simulations of the model for finite populations (see [10] and Fig. 3) and with the solution of the voter model when “zealots” (agents that are always in state 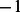) are present in the population [26], [27]. In the voter model, a small fraction of zealots is enough to throw the system into a negative efficiency value.

If the population contains a mixture of 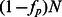 naive agents and 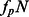 partisan agents with equal fractions of positive and negative partisans, then one recovers 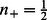 as a solution to Eq. (4). Note that in such case the steady-state solution 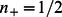 corresponds to 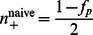, 
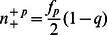
 and 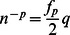, with 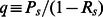 where 
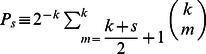
 and 
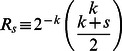
.

Similarly to the case of well-intentioned agents, one can assume that close to the transition the solution is 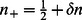 where 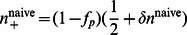, 
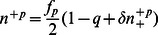
, and 
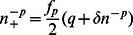
 to obtain the following transition line that separates efficient and non-efficient regimes (see Methods)
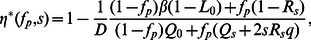
(14)where 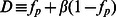 and 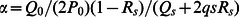. In fact, 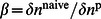 is the relative fraction of agents holding the majority opinion in excess of the stable solution for 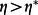. Note that 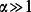
– actually 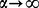 for 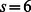, so that, close to the transition, the number of agents holding opinion 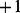 can only increase through the change of opinions of naive agents. Therefore, the introduction of both types of partisan agents has the opposite effect of the introduction of conservative agents. Whereas having some conservative agents results in a system whose efficiency in reaching consensus is more robust to noise, introducing partisan agents reduces the ability of the system to reach consensus as the noise amplitude increases. In fact, the larger the fraction of partisans and the stronger their bias strength, the lower the efficiency in reaching consensus as 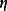 increases (Fig. 2).

## Discussion

Our work is the first to produce analytical results on the noise driven transition between efficient and non-efficient/partisan regimes when agents with bias are present in a model for consensus formation.

Seaver et al. [10] used numerical simulations to show the effect that including conservative or partisan agents in a population of naive agents has in the reaching of consensus in the absence of a central authority when noise is present. In here, we investigate the same model analytically in the mean-field approximation. We show that the mean-field solution can fully capture the transition from an efficient to an inefficient/partisan regime observed in numerical simulations of finite populations in [10]. Figure 4a compares the mean-field theoretical solution for the population efficiency to the average efficiency of a finite population with a finite number of neighbors in a Watts-Strogatz rewired network as in [10]. For most of the cases we study, we find that as the system size increases, the average efficiency approaches the mean-field solution, demonstrating that our results are a good approximation to numerical simulations of more realistic models for both the average efficiency values and the noise amplitude 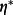 at which the change of regime takes place. Even for small population sizes one can observe the signatures of the transition both when conservatives and negative partisans are present. Note that in the case of a small initial majority 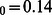, the average efficiency for 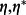 is lower than expected. This is because most configurations end up having a negative efficiency for 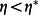 (Fig. 3b). If we take into account configurations that end up with a positive efficiency (Fig. 3c), we observe how the efficiency approaches the theoretical one as population size increases. In the other cases, this has no effect since for 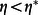 most configurations have a positive efficiency.

Interestingly, our calculations uncover the mechanisms that drive these transitions and show how the microscopic differences in agent bias relate to the macroscopic differences observed in the efficiency to reach consensus. Importantly, the results in Fig. 3 indicate that these mechanisms are also responsible for the transition observed in more realistic numerical simulations for finite populations. In the case of a system of naive and conservative agents, the fraction of conservative agents holding the majority opinion in excess of 1/2 (efficient regime) for 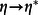 is larger than that of naive agents. As a consequence, as the fraction of conservative agents increases, the transition shifts to larger values of 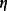, meaning that the ability of the system to reach consensus increases.

The inclusion of equal fractions of both types of partisans has the opposite effect. Since the relative fraction of naive agents holding the majority opinion in excess of 1/2 (efficient regime) for 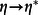 is much larger than that of partisan agents, as the fraction of partisans increases the system becomes less robust to noise and the transition shifts to smaller values of 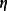. In fact, this last result highlights the importance of introducing naive agents in a biased population to guarantee a democratic outcome as it has been recently shown in communities of animals [29]. Our results thus add additional insights into the mechanisms behind opinion formation dynamics and could be useful for the future analysis of more complicated models.

## Methods

### Agents with no opinion bias: conservatives

Consider a mixed system of 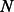 agents composed of 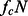 conservative agents with fixed bias strength 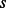 and 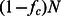 naive agents. To obtain the transition line 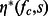 between efficient and non-efficient regimes, we assume that since the system is symmetric, the solution to Eq. (10) close to the transition for 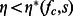 is 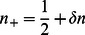 with 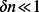. Without loss of generality we can assume that 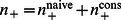 with 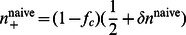 and 
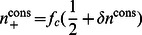
. Introducing these solutions into Eq. (10) for each type of agent we obtain the following expressions
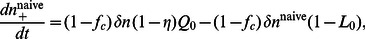
(15)

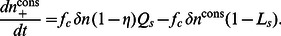
(16)


By imposing the steady-state condition on Eqs. (15) and (16), we find the relationship between 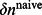 and 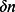, and 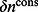 and 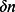, respectively. Noting that 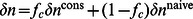, we obtain the following expression for Eq. (9)
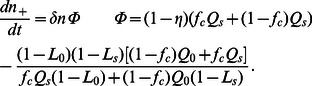
(17)Which setting 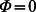 so that the steady state condition is fulfilled for all 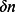 yields the expression for 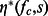 in Eq. (12). Note that by imposing the steady-state condition on Eqs. (15) and (16), we can also find the expression for 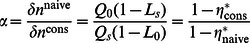 in Eq. (13).

### Agents with no opinion bias: both types of partisans

Consider a mixed population of 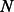 agents composed of 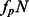 partisan agents with fixed bias strength 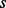 and equal fractions of negative and positive partisans, and 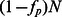 naive agents. As already explained in the main text, while this is a symmetric problem with a non efficient solution 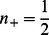, the relative fraction of the number of agents holding the majority opinion depends on the type of agent so that 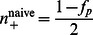, 
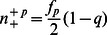
 and 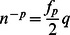, with 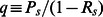 where 
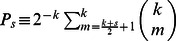
 and 
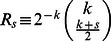
.

To obtain the transition line 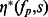 between efficient and non-efficient regimes, we thus assume that the solution to Eq. (10) close to the transition for 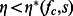 is 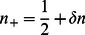 with 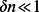. Without loss of generality, we can assume that 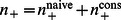 with 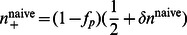, 
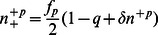
, and 
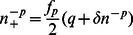
. Introducing these solutions into Eq. (10) for each type of agent we obtain the following expressions
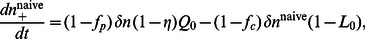
(18)

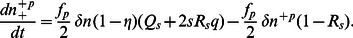
(19)

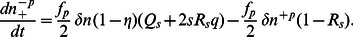
(20)


By imposing the steady-state condition on Eqs. (18-20), we find the relationship between 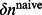 and 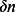, 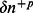 and 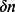 and 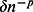 and 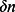, respectively. Note that because Eqs. (19) and (20) are the same, 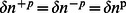. This means that the deviations from the steady state solution in which there are many more positive than negative partisans holding the majority opinion are the same for both types of partisans. Using that 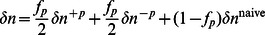, we obtain the following expression for Eq. (9)
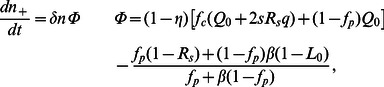
(21)where 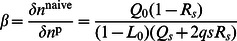. Setting 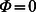 so that the steady state condition is fulfilled for all 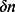, we obtain the expression for 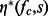 in Eq. (14).

## References

[1] Gelvin JL (2012) The Arab uprisings: what everybody needs to know. Oxford University Press.

[2] URL http://movimiento15m.org/.Accessed 2013 May 2.

[3] Surowiecki J (2004) The Wisdom of Crowds. Doubleday.

[4] Ball P (2004) Critical Mass: How One Thing Leads to Another. Farrar, Strauss & Giroux, New York.

[5] McCarty N, Poole KT, Rosenthal H (2006) Polarized America: The Dance of Ideology and Unequal Riches. MIT Press.

[6] Baldassarri D, Gelman A (2008) Partisans without constraint: Political polarization and trends in american public opinion. Am&$146;J&$146;Soc 114: 408–446. 10.2139/ssrn.1010098 PMC4056259 24932012

[7] Majumder SR, Diermeier D, Rietz TA, Amaral LAN (2009) Price dynamics in political prediction markets. Proc Natl Acad Sci&$146;U&$146;S&$146;A 106: 679–684. 10.1073/pnas.0805037106 PMC2630077 19155442

[8] Masuda N, Redner S (2011) Can partisan voting lead to truth? J&$146;Stat Mech: L02002.

[9] Moreira AA, Mathur A, Diermeier D, Amaral LAN (2004) Efficient system-wide coordination in noisy environments. Proc Natl Acad Sci USA 101: 12083–12090. 10.1073/pnas.0400672101 PMC514439 15297617

[10] Seaver SM, Moreira AA, Sales-Pardo M, Malmgren RD, Diermeier D, et al (2009) Micro-bias and macro-performance. Eur Phys&$146;J&$146;B 67: 369–375. 10.1140/epjb/e2008-00406-4 PMC4029510 24860255

[11] Liggett T (1985) Interacting Particle Systems. Springer.

[12] Castellano C, Fortunato S, Loreto V (2009) Statistical physics of social dynamics. Rev Mod Phys 81: 59–646.

[13] Castellano C, Vilone D, Vespignani A (2003) Incomplete ordering of the voter model on small-world networks. Europhys Lett 63: 153–158.

[14] Sood V, Redner S (2005) Voter model on heterogeneous graphs. Phys Rev Lett 94: 178701. 15904343 10.1103/PhysRevLett.94.178701

[15] Suchecki K, Eguíluz VM, Miguel MS (2005) Voter model dynamics in complex networks: Role of dimensionality, disorder, and degree distribution. Phys Rev&$146;E&$146;Stat Nonlin Soft Matter Phys 72: 036132. 10.1103/PhysRevE.72.036132 16241540

[16] Vazquez F, Eguíluz V (2008) Analytical solution of the voter model on uncorrelated networks. New&$146;J&$146;Phys 10: 063011.

[17] Lambiotte R, Ausloos M (2007) Coexistence of opposite opinions in a network with communities. J&$146;Stat Mech: Theor Exp: P08026.

[18] Lambiotte R, Ausloos M, Holyst JA (2007) Majority model on a network with communities. Phys Rev&$146;E&$146;Stat Nonlin Soft Matter Phys 75: 030101. 10.1103/PhysRevE.75.030101 17500655

[19] Krapivsky PL, Redner S (2003) Dynamics of majority rule in two-state interacting spin systems. Phys Rev Lett 90: 238701. 12857298 10.1103/PhysRevLett.90.238701

[20] Galam S, Jacobs F (2007) The role of inflexible minorities in the breaking of democratic opinion dynamics. Physica&$146;A 381: 366–376.

[21] Lambiotte R, Redner S (2008) Dynamics of non-conservative voters. Europhys Lett 82: art. no. 18007: 1–5.

[22] Galam S (2009) Sociophysics: A review of Galam models. Int&$146;J&$146;Mod Phys&$146;C 19: 409–440.

[23] Hegselmann R, Krause U (2002) Opinion dynamics and bounded confidence: models, analysis and simulation. Journal of Artificial Societies and Social Simulation 5: art. num. 2.

[24] Fortunato S (2004) The Krause-Hegselmann consensus model with discrete opinions. Int&$146;J&$146;Mod Phys&$146;C 15: 1021.

[25] Campos PRA, de Oliveira VM, Moreira FGB (2003) Small-world effects in the majority-vote model. Phys Rev E 67: art. no. 026104. 10.1103/PhysRevE.67.026104 12636745

[26] Mobilia M (2003) Does a single zealot affect an infinite group of voters? Phys Rev Lett 91: 028701. 12906515 10.1103/PhysRevLett.91.028701

[27] Mobilia M, Petersen A, Redner S (2007) On the role of zealotry in the voter model. J&$146;Stat Mech: Theor Exp: P08029.

[28] Volovik D, Redner S (2012) Dynamics of confident voting. J&$146;Stat Mech.

[29] Couzin ID, Ioannou CC, Demirel G, Gross T, Torney CJ, et al (2011) Uninformed individuals promote democratic consensus in animal groups. Science 334: 1578. 22174256 10.1126/science.1210280

[30] Watts DJ, Strogatz SH (1998) Collective dynamics of ‘small-world’ networks. Nature 393: 440–442. 9623998 10.1038/30918

